# Early career psychiatrists’ perceptions of and training experience in electroconvulsive therapy: A cross-sectional survey across Europe

**DOI:** 10.1192/j.eurpsy.2024.1798

**Published:** 2025-01-13

**Authors:** Cristiana Țăpoi, Laith Alexander, Renato de Filippis, Agorastos Agorastos, Diogo Almeida, Gautam Bhatia, Gamze Erzin, Martyna Ewa Gołębiewska, Enita Metaj, Sara Medved, Krista Mieze, Miloš Milutinović, Camille Noël, Antonina Pushko, David Gurrea Salas, Alejandro Compaired Sanchez, Alina Wilkowska, Rick P. F. Wolthusen, Mariana Pinto da Costa

**Affiliations:** 1 Department of General Psychiatry, Alexandru Obregia Clinical Psychiatry Hospital, Bucharest, Romania; 2 Institute of Psychiatry, Psychology & Neuroscience, King’s College London, London, UK; 3 Psychiatry Unit, Department of Health Sciences, University Magna Graecia of Catanzaro, Catanzaro, Italy; 4 II. Department of Psychiatry, School of Medicine, Faculty of Health Sciences, Aristotle University of Thessaloniki, Thessaloniki, Greece; 5 Department of Psychiatry and Mental Health, Hospital de Loures, Loures, Portugal; 6 South London and Maudsley NHS Foundation Trust, London, UK; 7 Department of Psychiatry, Dışkapı Yıldırım Beyazıt Training and Research Hospital, University of Health Sciences, Ankara, Turkey; 8 Department of Psychiatry and Neuropsychology, School for Mental Health and Neuroscience, Maastricht University Medical Centre, Maastricht, the Netherlands; 9 Department of Developmental, Psychotic, and Geriatric Psychiatry, Medical University of Gdańsk, Gdańsk, Poland; 10 Community Mental Health Center No. 2, Tirana, Albania; 11 Department of Psychiatry and Psychological Medicine, University Hospital Centre Zagreb, Zagreb, Croatia; 12 Department of Doctoral Studies, Riga Stradins University, Riga, Latvia; 13 University Clinic of Psychiatry, Skopje, North Macedonia; 14 Department of Psychiatry, Centre Hospitalier Universitaire Saint-Pierre, Université Libre de Bruxelles, Brussels, Belgium; 15 Department of Psychiatry, Narcology and Medical Psychology Ivano-Frankivsk National Medical University, Communal Non-Commercial Enterprise Precarpathian Regional Clinical Center for Mental Health of the Ivano-Frankivsk Regional Council, Ivano-Frankivsk, Ukraine; 16 Department of Addictive Disorders, Psychiatric Services Aargau, Brugg, Switzerland; 17 Department of Psychiatry, Ramon y Cajal University Hospital, Madrid, Spain; 18 Department of Psychiatry, Medical University of Gdańsk, Gdańsk, Poland; 19 Department of Psychiatry and Behavioral Sciences, Duke University Medical Center, Durham, NC, USA; 20 Division of Psychotic Disorders, McLean Hospital, Belmont, MA, USA; 21 Institute of Biomedical Sciences Abel Salazar, University of Porto, Porto, Portugal

**Keywords:** cross-sectional studies, electroconvulsive therapy, electroshock, Europe, psychiatry, training

## Abstract

**Background:**

Electroconvulsive therapy (ECT) is a safe and effective treatment for several major psychiatric conditions, including treatment-resistant depression, mania, and schizophrenia; nevertheless, its use remains controversial. Despite its availability in some European countries, ECT is still rarely used in others. This study aims to investigate the experiences and attitudes of early career psychiatrists (ECPs) across Europe towards ECT and to examine how their exposure to ECT influences their perceptions.

**Methods:**

In Europe, a cross-sectional survey was conducted among ECPs, including psychiatric trainees and recently fully qualified psychiatrists.

**Results:**

A total of 573 participants from 30 European countries were included in the study, of whom more than half (*N* = 312; 54.5%) received ECT training. Overall, ECPs had a positive attitude towards ECT, with the vast majority agreeing or strongly agreeing that ECT is an effective (*N* = 509; 88.8%) and safe (*N* = 464; 81.0%) treatment and disagreeing or strongly disagreeing that ECT was used as a form of control or punishment (*N* = 545; 95.1%). Those who had received ECT training during their psychiatry training were more likely to recommend ECT to their patients (p < 0.001, r = 0.34), and held more positive views on its safety (p < 0.001, r = 0.31) and effectiveness (p < 0.001, r = 0.33). Interest in further education about ECT was moderately high (modal rating on Likert scale: 4, agree), irrespective of prior training exposure.

**Conclusions:**

ECT training is associated with more favorable perceptions of its safety and effectiveness among ECPs. There is a general willingness among ECPs to expand their knowledge and training on ECT, which could enhance patients’ access to this treatment.

## Introduction

Electroconvulsive therapy (ECT) was first used in 1938 by Ugo Cerletti and Lucio Bini to treat schizophrenia. The historical origins of convulsive treatment can be traced back to the 16^th^ century, and prior to the use of electric stimuli, seizure-inducing agents like camphor, insulin, and metrazol were used in psychiatric practice [1,2]. The introduction of ECT filled a critical gap in psychiatric care, as no other interventions were available at the time for severe mental disorders [3,4]. Over 80 years later, ECT remains a crucial treatment in psychiatry, with approximately 1.4 million treatment courses administered annually worldwide [5–7].

The UK NICE guidelines recommend ECT for severe cases of depression in bipolar or unipolar depressive disorders, schizophrenia, catatonia, and prolonged or severe manic episodes [8]. While typically considered after pharmacotherapy has failed, ECT is also appropriate as an initial treatment in life-threatening situations, such as suicidal intent, severe agitation, or refusal of food and fluids, where rapid symptom relief is crucial [9,10].

ECT is a safe procedure, performed under general anesthesia, and with muscle relaxants [11–19]. It has a very low mortality rate (2.1 per 100,000 treatments) and no absolute contraindications [5,11,20,21]. The side effect often of most concern to patients, retrograde amnesia – particularly affecting autobiographical memories – can be mitigated by using unilateral electrode placement and brief or ultra-brief pulse stimulation, making it less common and often transient [5,11,21,22].

Despite the scientific evidence for its efficacy, ECT remains controversial among patients, caregivers, and some psychiatrists [20,23]. This reluctance is often fueled by negative media portrayals, lack of knowledge about advances in ECT techniques, concerns over side effects, stigma, and limited access to specialized centers [20,24–27]. Cross-sectional studies dating from the 1980s to the 2010s indicated increased recognition of ECT’s efficacy among psychiatrists, reflected in more positive attitudes and better knowledge compared to other healthcare professionals, such as nurses, psychologists, or general practitioners [28–37]. However, some psychiatrists were dissatisfied with the training they received and wanted a more comprehensive theoretical and practical education [38–41].

Research shows that ECT availability has increased in Western European countries, such as Germany, Belgium, and Spain, but remains limited in Central-Eastern Europe [20,24,42–46]. A systematic review published in 2012 [7] highlighted the global variability in ECT use, with rates ranging from 0.11 patients per 10,000 inhabitants per year in Poland, 0.26 in Germany, 0.31 in Hungary, 0.61 in Spain, 4.27 in Belgium, and 5.10 in the United States of America. Recent national surveys also report varying rates of ECT-treated individuals across European countries, with 0.04 per 10,000 inhabitants per year in Italy [27], 0.13 in Poland [47], 0.48 in Switzerland [48], 0.49 in France [49], 0.69 in Germany [50] and 1.36 in the Czech Republic [51]. Regarding the use of ECT in children and adolescents in Europe, a literature review [52] published in 2023 found that, despite its inconsistent utilization, ECT is effective and not linked to any long-term side effects in children and adolescents. This uneven distribution of ECT use may be explained by political, economic, and cultural factors [42,43].

In this evolving landscape, understanding the perspectives of early career psychiatrists (ECPs) regarding ECT is essential. Evidence from cross-sectional surveys suggests that negative preconceptions about ECT were reversed in medical students who had the opportunity to interact with ECT experts and see the procedure in real-life [53–56]. However, little is known about ECPs’ experiences with ECT and how their opinions about ECT are shaped.

This study aims to assess the experiences and attitudes of ECPs in Europe towards ECT, and how their access to ECT training opportunities influences their perceptions.

## Methods

### Study design

A cross-sectional study was conducted using an online questionnaire. The study received approval from the Ethics Committee of the Alexandru Obregia Clinical Psychiatry Hospital in Bucharest, Romania (no. 84/10.03.2022).

### Study instrument

The questionnaire was developed in English and comprised 36 single-select multiple-choice questions and 5-point Likert scale questions (1 = strongly disagree; 5 = strongly agree). These questions covered participants’ socio-demographic characteristics, professional background, the availability of ECT in their institution or within 100 km of their workplace, experience with ECT, receipt of ECT training during psychiatry training, and attitudes and knowledge about ECT.

### Data collection

The online questionnaire was distributed to ECPs between July 2022 and July 2024 by email or through international social media groups (e.g. Facebook or WhatsApp) to ECPs. Participants were encouraged to disseminate the questionnaire among their peers.

Inclusion criteria required participants to be working in a European country and to be an ECP. The definition of ECP includes psychiatric trainees and psychiatrists who completed their psychiatry training within the last 5 years.

### Data analysis

Statistical analysis was performed using the Software Package for Social Sciences v. 29.0.2.0 (SPSS Inc.). Descriptive statistical data are presented as counts, mean values, standard deviations, and percentages. Wilcoxon Signed Rank Sum test, Student’s t-test, and Chi-Square test were used to compare subgroups. A p-value of less than 0.05 was considered statistically significant.

## Results

### Sample characteristics

Demographic data are summarized in Table 1. A total of 655 participants from 44 countries responded to the questionnaire. However, 25 participants who were not working in Europe and 57 participants who exceeded 5 years of completing their psychiatric training were excluded. The final sample comprised 573 ECPs from 30 European countries: Albania, Belgium, Bulgaria, Croatia, Czech Republic, Finland, France, Germany, Greece, Hungary, Iceland, Ireland, Italy, Latvia, Luxembourg, Malta, Moldova, North Macedonia, Poland, Portugal, Romania, Russia, Slovakia, Spain, Sweden, Switzerland, The Netherlands, Turkey, United Kingdom, and Ukraine.

**Table 1.** Demographic characteristics of the sample

		Received ECT training		Group difference		
				**p-value**	**t-value**	**df**
	**Total** (*N* = 573;100%)	**Yes** (*N* = 312;54.5%)	**No** (*N* = 261;45.5%)			

*Age*				0.007	2.715	571
	31.83 (SD 4.980)	32.35 (SD 5.092)	31.22 (SD 4.780)			
				**p-value**	**χ2**	**df**

*Gender*				0.044	6.229	2
Woman	374 (65.3%)	191 (61.2%)	183 (70.1%)			
Man	193 (33.7%)	116 (37.2%)	77 (29.5%)			
Non-binary	6 (1.0%)	5 (1.6%)	1 (0.4%)			

*Country*				<0.001	295.801	29
United Kingdom	105 (18.3%)	102 (32.7%)	3 (1.1%)			
Poland	93 (16.2%)	59 (18.9%)	34 (13.0%)			
Romania	79 (13.8%)	21 (6.7%)	58 (22.2%)			
Italy	52 (9.1%)	1 (0.3%)	51 (19.5%)			
Greece	42 (7.3%)	5 (1.6%)	37 (14.2%)			
Portugal	29 (5.1%)	25 (8.0%)	4 (1.5%)			
Germany	28 (4.9%)	22 (7.1%)	6 (2.3%)			
Spain	25 (4.4%)	21 (6.7%)	4 (1.5%)			
Latvia	20 (3.5%)	0 (0%)	20 (7.7%)			
Albania	19 (3.3%)	4 (1.3%)	15 (5.7%)			
Belgium	17 (3.0%)	12 (3.8%)	5 (1.9%)			
Turkey	10 (1.7%)	10 (3.2%)	0 (0.0%)			
Switzerland	8 (1.4%)	1 (0.3%)	7 (2.7%)			
Other	46 (8.0%)	29 (9.3%)	17 (6.5%)			

*Main workplace*				0.232	10.491	8
Inpatient Center	304 (53.1%)	153 (49.0%)	151 (57.9%)			
Outpatient Center	213 (37.2%)	123 (39.4%)	90 (34.5%)			
Private practice	28 (4.9%)	17 (5.4%)	11 (4.2%)			
Research Center	16 (2.8%)	11 (3.5%)	5 (1.9%)			
Liaison	7 (1.2%)	5 (1.6%)	2 (0.8%)			
Other	5 (0.8%)	3 (0.9%)	2 (0.8%)			

*Working field*				0.109	7.566	4
Adult psychiatry	487 (85.0%)	260 (83.3%)	227 (87.0%)			
Child/adolescent psychiatry	48 (8.4%)	24 (7.7%)	24 (9.2%)			
Elderly psychiatry	25 (4.4%)	20 (6.4%)	5 (1.9%)			
Forensic psychiatry	7 (1.2%)	4 (1.3%)	3 (1.1%)			
Medical psychotherapy	6 (1.0%)	4 (1.3%)	2 (0.8%)			

*Professional experience*				0.001	14.916	2
1st or 2nd year psychiatric trainee	181 (31.6%)	78 (25.0%)	103 (39.5%)			
3rd year psychiatric trainee or more	219 (38.2%)	136 (43.6%)	83 (31.8%)			
Psychiatrist who finished training in the last 5 years	173 (30.2%)	98 (31.4%)	75 (28.7%)			

Most respondents (*N* = 374; 65.3%) were women, mostly from the United Kingdom (*N* = 105; 18.3%), Poland (*N* = 93; 16.2%), and Romania (*N* = 79; 13.8%). The majority (*N* = 400; 69.8%) were psychiatric trainees at the time of completing the questionnaire, while the remainder (*N* = 173; 30.2%) were psychiatrists who had completed their training within the last 5 years. Most respondents (*N* = 487; 85.0%) worked as adult psychiatrists, with over half (*N* = 304; 53.1%) working in an inpatient mental health center. The vast majority (*N* = 531; 92.7%) reported that ECT was available in their country, and over half (*N* = 295; 51.5%) were working in an institution where ECT was available. Some respondents (*N* = 102; 17.8%) stated that an ECT clinic was not available within 100 km of their workplace, while a few (*N* = 38; 6.6%) were unaware of the presence of such a clinic.

### ECT training

Over half of the respondents (*N* = 312; 54.5%) received ECT training during their psychiatry training, while the remainder (*N* = 261; 45.5%) did not. ECT training included clinical rotations focused on ECT (*N* = 147; 47.1%), didactic ECT teaching, such as lectures or workshops focused on ECT (*N* = 91; 29.1%), practical experience through shadowing ECT sessions or informal learning (*N* = 61; 19.5%), or a combination of both practical and didactic ECT training (*N* = 13; 4.1%). Most respondents (*N* = 228; 73.1%) who received ECT training participated in administering ECT treatments under the supervision of another psychiatrist, while some (*N* = 61; 19.5%) administered ECT treatments without supervision. The mean age was significantly lower among those who did not receive ECT training (31.22; SD 4.78 versus 32.35; SD 5.09, p-value = 0.007). Significant differences in gender, professional experience, and country of employment were also observed between the two groups.

### Overall perceptions of ECT

**Figure 1.**
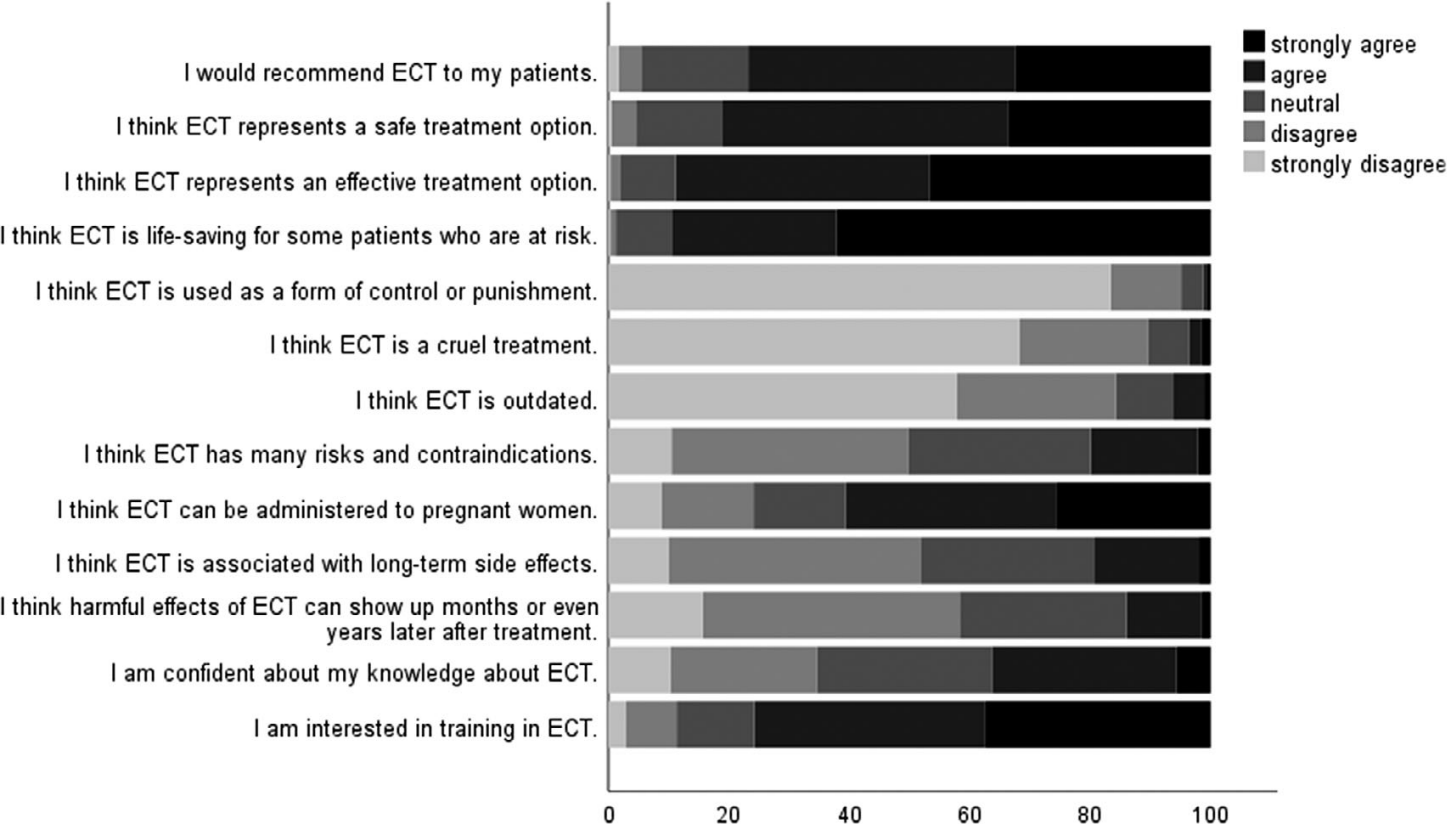
Perception of ECT among European ECPs in our sample.

The perceptions of ECPs towards ECT across the entire sample are shown in Figure 1 and Table 2. Most ECPs (*N* = 440; 76.8%) agreed or strongly agreed that they would recommend ECT to their patients, and the majority agreed or strongly agreed that ECT was an effective (*N* = 509; 88.8%) and safe (*N* = 464; 81.0%) treatment. The majority agreed or strongly agreed that ECT can be lifesaving (*N* = 513; 89.5%).

**Figure 2.**
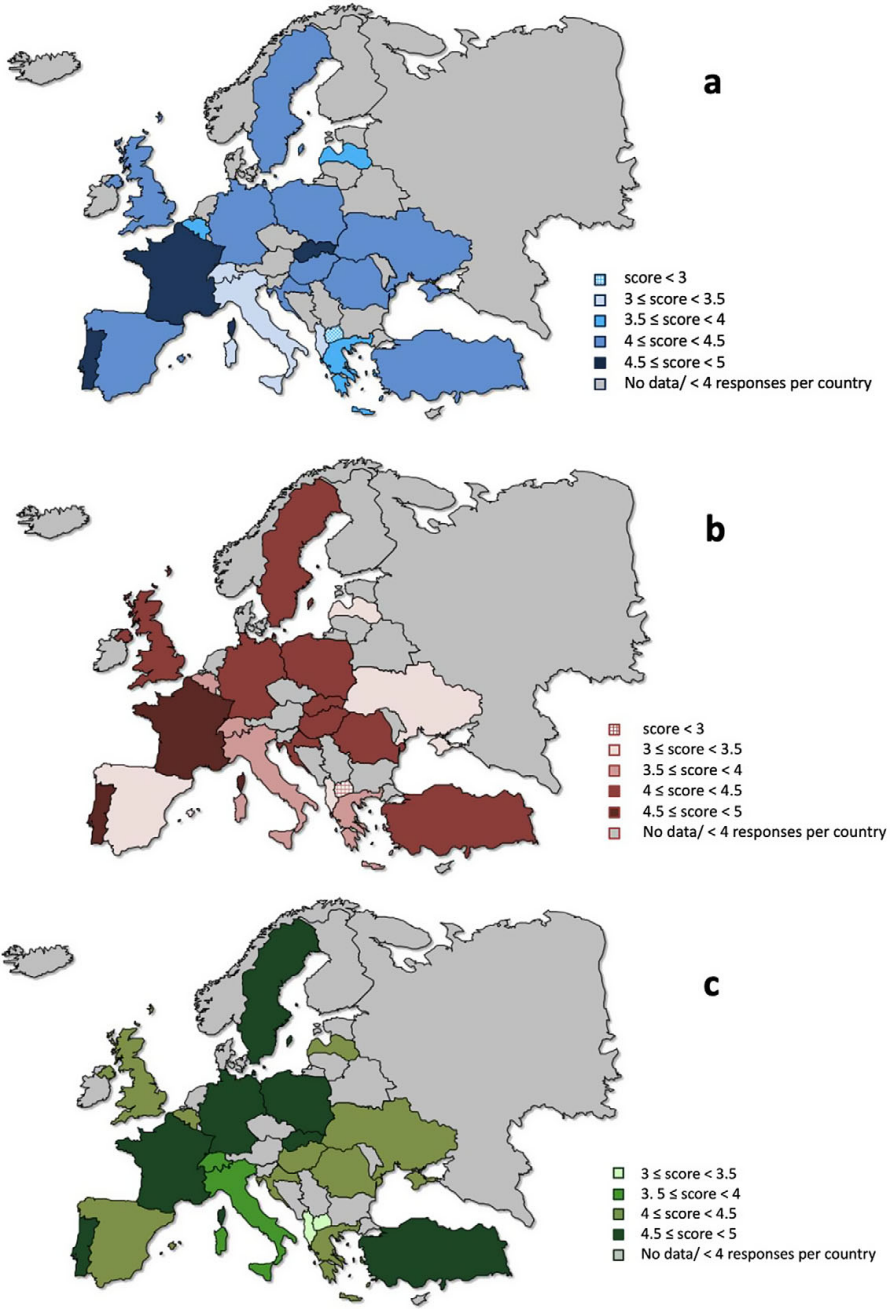
Maps of Europe reporting mean responses to the following statements: **a.** “I would recommend ECT to my patients.”; **b.** ‘I think ECT represents a safe treatment option.’; **c.** ‘I think ECT represents an effective treatment option.’

**Table 2.** Differences in attitudes and knowledge about ECT in psychiatrists who did and did not receive ECT training

		**Received ECT training**				
	**Total (*N* = 573;100%)**	**Yes (*N* = 312;54.5%)**	**No (*N* = 261;45.5%)**	**p** **-value**	**Mean difference [95% CI]**	**Effect size (r)**
I would recommend ECT to my patients.	4.02 (SD 0.898)	4.29 (SD 0.771)	3.69 (SD 0.931)	p < 0.001	0.60 [0.46–0.74]	0.347
I think ECT represents a safe treatment option.	4.09 (SD 0.825)	4.33 (SD 0.710)	3.82 (SD 0.866)	p < 0.001	0.51 [0.38–0.64]	0.312
I think ECT represents an effective treatment option.	4.33 (SD 0.742)	4.54 (SD 0.630)	4.07 (SD 0.784)	p < 0.001	0.47 [0.35–0.59]	0.329
I think ECT is lifesaving for some patients who are at risk.	4.50 (SD 0.728)	4.79 (SD 0.490)	4.16 (SD 0.816)	p < 0.001	0.62 [0.51–0.73]	0.444
I think ECT is used as a form of control or punishment.	1.23 (SD 0.597)	1.14 (SD 0.448)	1.34 (SD 0.724)	p < 0.001	0.19 [0.09–0.29]	0.153
I think ECT is a cruel treatment.	1.47 (SD 0.827)	1.33 (SD 0.715)	1.64 (SD 0.916)	p < 0.001	0.31 [0.17–0.44]	0.204
I think ECT is outdated.	1.65 (SD 0.922)	1.46 (SD 0.789)	1.88 (SD 1.014)	p < 0.001	0.42 [0.28–0.57]	0.245
I think ECT has many risks and contraindications.	2.62 (SD 0.964)	2.40 (SD 0.967)	2.88 (SD 0.893)	p < 0.001	0.48 [0.33–0.64]	0.258
I think ECT can be administered to pregnant women.	3.53 (SD 1.266)	3.88 (SD 1.129)	3.12 (SD 1.301)	p < 0.001	0.75 [0.55–0.95]	0.295
I think ECT is associated with long-term side effects.	2.59 (SD 0.950)	2.55 (SD 1.016)	2.64 (SD 0.863)	p = 0.207	0.09 [−0.06–0.25]	0.052
I think harmful effects of ECT can show up months or even years after treatment.	2.41 (SD 0.945)	2.34 (SD 1.014)	2.51 (SD 0.849)	p = 0.010	0.17 [0.01–0.32]	0.108
I am confident about my knowledge about ECT.	2.97 (SD 1.090)	3.47 (SD 0.889)	2.36 (SD 0.997)	p < 0.001	1.11 [0.95–1.26]	0.509
I am interested in training in ECT.	3.99 (SD 1.052)	3.97 (SD 1.064)	4.00 (SD 1.040)	p = 0.806	0.03 [−0.12–0.20]	0.010
				**p-value**	**χ2 value**	**df**
Awareness of national ECT guidelines	330 (57.6%)	231 (74.0%)	99 (37.9%)	p < 0.001	75.862	1
Awareness of international ECT guidelines	87 (15.2%)	54 (17.3%)	33 (12.6%)	p = 0.121	2.401	1

Most respondents disagreed or strongly disagreed that ECT was used as a form of control or punishment (*N* = 545; 95.1%), that ECT was outdated (*N* = 483; 84.3%), or that ECT was a cruel treatment (*N* = 513; 89.5%). However, perceptions of risks, contraindications, and side effects were somewhat more mixed, with 114 (19.9%) agreeing or strongly agreeing that ECT had many risks and contraindications. Several respondents (*N* = 110; 19.2%) agreed or strongly agreed that ECT was associated with long-term side effects or believed these could emerge months or years after treatment (*N* = 80; 14.0%). Most (*N* = 347; 60.6%) agreed or strongly agreed that ECT could be administered to pregnant women. Several (*N* = 208; 36.30%) agreed or strongly agreed they are confident in their knowledge of ECT. The majority of trainees indicated they would be interested in further ECT training (*N* = 434; 75.7% agreed or strongly agreed). The opinions on ECT varied across European countries, with a higher willingness to recommend ECT in participants from Portugal, Spain, the United Kingdom, Germany, and Poland (mean score greater than or equal to 4), and a lower willingness to do so (mean score less than 4) in countries such as Italy, Albania or Latvia. Moreover, participants from Portugal, the United Kingdom, Spain, Germany, Poland, Belgium, and Romania agreed or strongly agreed that ECT is a safe and effective treatment option (mean score greater than or equal to 4), compared to participants from Albania, Italy, North Macedonia (mean score less than 4). The mean values of the distributions of opinions for 3 of the items in the questionnaire, calculated for each country, are illustrated in Figure 2.

### Effect of training on perception

The effects of ECT training on perceptions are highlighted in Table 2. Psychiatrists who had undergone ECT training displayed a more positive attitude towards safety (mean difference 0.51; 95% CI 0.38–0.64, p < 0.001) and effectiveness (mean difference 0.47; 95% CI 0.35–0.58, p < 0.001) of ECT and seemed more willing to recommend ECT to their patients (mean difference 0.60; 95% CI 0.46–0.74, p < 0.001) compared to those psychiatrists who did not receive training. ECPs who were exposed to ECT training were more confident in their knowledge of the procedure (mean difference 1.11; 95% CI 0.95–1.26, p < 0.001) and were more aware of the fact that ECT can be safely used during pregnancy (mean difference 0.75; 95% CI 0.55–0.95, p < 0.001). Respondents who received ECT training were less likely to consider that ECT was a cruel treatment (mean difference 0.31; 95% CI 0.17–0.44, p < 0.001), or to believe that it was currently used abusively (mean difference 0.19; 95% CI 0.09–0.29, p < 0.001). Demographic differences between groups had no significant effect on perceptions of ECT.

## Discussion

### Key findings

Overall, ECPs had a positive attitude towards ECT. The majority agreed or strongly agreed they would recommend ECT to their patients (76.8%), that ECT is an effective and safe treatment (88.8 and 81.0%, respectively), and disagreed or strongly disagreed that ECT was used as a form of control or punishment (95.1%) or that ECT was outdated (84.3%). Just over half of the respondents (54.5%) had access to ECT training during their psychiatry training. Those who received ECT training had a more positive attitude regarding the safety and effectiveness of ECT and were more willing to recommend ECT to their patients, compared to those who had not. All respondents showed an interest in receiving further ECT training.

### Comparison with the other literature

Psychiatrists in our study group had a favorable opinion about ECT, a finding which is consistent with the results from previous European, American, Saudi Arabian, and South African surveys [29,33,36,37,39,57]. For example, a cross-sectional survey in London, UK [29] that evaluated attitudes towards and knowledge of ECT in mental health professionals (psychiatrists, psychologists, nurses, and social workers) revealed that 83% of the psychiatrists believed that ECT is more beneficial than harmful, 79% believed that ECT is unlikely to cause brain damage, 91% disagreed on ECT being a cruel treatment, and 87% agreed that ECT was effective in treating depression. Conversely, a cross-sectional survey that was performed on a sample of 40 Romanian psychiatrists [35] showed a more negative view of ECT, with 65% considering ECT outdated and 62.5% believing it is used to control violent patients. These differing attitudes may reflect the variations in access to ECT training.

Our findings also demonstrated that psychiatrists who had access to ECT training had a more positive attitude towards its safety and effectiveness, and were more knowledgeable about its indications in pregnancy and about the low-risk profile of the procedure. The relation between exposure to the practice of ECT and a more favorable perception of ECT is supported by a survey in the United States of America conducted by psychiatrists employed in state hospitals in Texas [36]. Similar findings were revealed by studies with medical students before and after ECT education training [53,58,59]. Two multi-group comparative studies in which a questionnaire was administered to psychiatrists, psychologists, nurses, and social workers [28,33] found that the level of knowledge about ECT directly influences mental health practitioners’ attitude towards ECT and hence the likelihood of recommending ECT to their patients.

While greater knowledge of ECT is associated with a more positive attitude towards ECT among psychiatrists [30,33,37,60], studies have shown that even when psychiatrists have a positive attitude regarding ECT, they are sometimes dissatisfied with the training received [38–41,56]. In our study, most respondents were interested in gaining more knowledge on ECT and having the chance to get more hands-on training, even though those exposed to ECT training were more confident in their knowledge of the topic. This might relate to the different types of ECT education received by our respondents, which varied substantially. Some programs included clinical rotations focused on ECT (46.4%), other courses or workshops focused on ECT (29.7%), or a combination of practical and didactic teaching (3.9%). However, 19.9% of those who received ECT training described it as merely shadowing ECT sessions or informal learning. According to the American Psychiatric Association (APA) [10], modern ECT requires thorough practitioner training in stimulus dosing, electrode placement, pharmacological interactions, and monitoring during the procedure, as well as pre- and post-stimulus administration. The APA recommends that ECT training during residency programs should include both practical training experience and formal teaching sessions [10]. Therefore, merely shadowing ECT sessions is insufficient to meet the minimal ECT training requirements, and lectures alone are inadequate to provide the necessary expertise for administering ECT.

There were notable discrepancies in access to ECT training across European countries. For example, there were countries in which most of the respondents received ECT education during residency training (United Kingdom, Portugal, Germany, Spain), while in other countries access to ECT training was less prominent (Romania, Greece, Albania, Latvia, Italy). These differences are in line with the reports showing a decreased use of ECT and fewer ECT centers in Central-Eastern Europe, compared to Western European countries [20,24,28–32]. What is more, participants from countries in which ECT training was available (United Kingdom, Portugal, Germany, Spain) were more likely to recommend ECT to their patients and had a more favorable opinion regarding the safety and effectiveness of ECT, compared to countries where access to ECT training was lower.

In our study, about half of the respondents (57.6%) were familiar with national ECT guidelines, while only a few (15.2%) were aware of international ECT guidelines, a finding that is indicative of the need for ECPs to supplement their expertise in ECT. This indicates a need for ECPs to enhance their expertise in ECT practices, as the lack of familiarity with national or international guidelines correlates with an insufficient standardization of ECT procedures and practices. A cross-sectional study published in 2022 [61] highlighted the need for standardized ECT guidelines to ensure ensuring treatment availability and utilization across different regions. The results of this study indicate high variability of the pre-ECT evaluation practice in 16 European countries, with slightly more than half of the countries having national regulations on pre-ECT evaluation, only half of the clinics using psychiatric scales, and just one-third of the clinics performing a cognitive assessment.

### Strengths and limitations

This is the first study investigating the experiences of European ECPs with ECT, providing valuable insights from countries with diverse economic backgrounds and training opportunities.

However, there are limitations to this study. The reliance on retrospective self-reports might lead to results that differ from direct observation of behaviors. Furthermore, the recruitment process may have attracted ECPs with a particular interest in ECT, leading to a selection bias. Additionally, the sample size is not large enough to allow drawing definite conclusions that are representative of the entire population, particularly when comparing countries with different economic backgrounds.

### Implication of the findings for future practice, policies, and research

Our findings highlight the disparities in ECT use and ECT training opportunities across European countries, as well as the significant variation in the types of ECT training available during psychiatric training programs. Three key factors influence clinicians’ decisions to prescribe ECT for patients suffering from major psychiatric disorders: a thorough understanding of ECT management and its potential benefits, exposure to ECT during psychiatry training, and a higher level of understanding of ECT’s safety and tolerability.

As ECT continues to play a crucial role in psychiatry, it is essential for all psychiatrists to be well informed on this topic. While not all psychiatrists will work in a center where ECT is performed, all psychiatrists should be knowledgeable enough about ECT to identify cases where it is indicated, provide comprehensive guidance to patients, and to make informed referrals to ECT centers.

In line with the needs expressed by our respondents regarding ECT learning, we advocate for systematic efforts to enhance ECT knowledge and training within the psychiatry training curriculum. Integrating theoretical knowledge with practical experience may increase clinicians’ confidence in addressing patients’ queries and recommending ECT when appropriate.

ECPs were our target population, as we aimed to capture the perspectives of those whose training reflects modern ECT techniques and who have not been exposed to the ‘unmodified’ form of ECT, an experience that could have affected their current opinions about ECT, potentially leading to biases based on outdated practices. Moreover, we focused on this group in order to explore whether ECPs were interested in pursuing additional training in ECT. Understanding ECPs’ attitudes towards ECT could help guide decisions about its future use and incorporation into training programs. Assessing their willingness to seek further education in ECT could highlight gaps in current psychiatric training and inform future curriculum development.

## Conclusions

ECPs generally have an overall positive attitude towards ECT. Access to ECT training during psychiatry training is associated with a greater likelihood of supporting its safety and effectiveness. However, there are still areas where ECT is inaccessible to patients and where psychiatrists do not receive proper education training. ECPs are willing to expand their knowledge and training on ECT, which could increase its accessibility and use in cases where it might significantly benefit the patients.

## Data Availability

The data supporting the conclusions of this paper will be made available upon request.
